# Impact of topical nifedipine on wound healing in animal model (pig)

**DOI:** 10.1590/1677-5449.190092

**Published:** 2020-07-06

**Authors:** Augusto Cézar Lacerda Brasileiro, Dinaldo Cavalcanti de Oliveira, Pollianne Barbosa da Silva, João Kairo Soares de Lima Rocha

**Affiliations:** 1 Faculdade de Medicina Nova Esperança – FAMENE, João Pessoa, PB, Brasil.; 2 Universidade Federal de Pernambuco – UFPE, Hospital das Clínicas, Recife, PE, Brasil.

**Keywords:** wound, topical nifedipine, polymorphonuclear cells, vascular proliferation, collagens, ferida, nifedipina tópica, células polimorfonucleares, proliferação vascular, colágeno

## Abstract

**Background:**

The human skin is an extremely sophisticated and evolved organ that covers the whole body. External agents or the patient’s own diseases can cause skin injuries that can challenge healthcare professionals and impose high social, economic and emotional costs.

**Objectives:**

To evaluate the impact of topical nifedipine on skin wound healing, specifically on polymorphonuclear cells, vascular proliferation, and collagen.

**Methods:**

We used three pigs, and created eight injuries in the dorsal region of each animal. We applied 1%, 10%, and 20% concentration nifedipine creams to four of the wounds in animals 1, 2, and 3 respectively and treated the other twelve wounds with saline solution 0.9% only. We analyzed the presence of polymorphonuclear cells, vascular proliferation, and collagen at six different times (days 1, 3, 7, 14, 21, and 28).

**Results:**

The evaluation of polymorphonuclear levels showed mild cell activity at all times in the control group, while in the nifedipine groups, marked levels were more frequent at all times during the experiment. There was a 4.84-fold increase in the chance of marked vascular proliferation (p = 0.019) and, at the same time, a decrease in collagen formation (OR 0.02 / p = 0.005) in animal 3.

**Conclusions:**

Topical NFD may have an impact on skin wound healing mechanisms. Our study showed that polymorphonuclear cells and vascular proliferation increased. We also demonstrated that collagen formation decreased. Therefore, topical NFD may have a positive impact on skin wound healing. Additional studies are needed to confirm our results.

## Resumo

**Contexto:**

A pele humana é um órgão extremamente sofisticado e evoluído que cobre todo o corpo. As lesões cutâneas podem ser causadas por agentes externos ou pelas próprias doenças do paciente, e podem representar um desafio para os profissionais de saúde com altos custos sociais, econômicos e emocionais.

**Objetivos:**

Avaliar o impacto da nifedipina tópica na cicatrização de feridas cutâneas, especialmente em relação a polimorfonucleares, proliferação vascular e colágeno.

**Métodos:**

Utilizamos três porcos e realizamos oito ferimentos na região dorsal de cada animal. Aplicamos as concentrações de nifedipina creme a 1%, 10% e 20% para os animais 1, 2 e 3, respectivamente, sendo que, em quatro ferimentos, aplicamos o creme e, nos outros quatro ferimentos, apenas soro fisiológico a 0,9%. Analisamos a presença de polimorfonucleares, proliferação vascular e colágeno em seis momentos diferentes (dias 1, 3, 7, 14, 21 e 28).

**Resultados:**

A avaliação dos níveis polimorfonucleares mostrou atividade celular discreta em todos os momentos no grupo controle, enquanto nos grupos nifedipina, os níveis marcados foram mais frequentes em todos os momentos do experimento. Houve aumento de 4,84 vezes na chance de uma produção marcada (p = 0,019) da proliferação vascular e, ao mesmo tempo, diminuição da formação do colágeno (*odds ratio,* OR 0,02; p = 0,005) no animal 3.

**Conclusões:**

A nifedipina tópica pode ter impacto no mecanismo de cicatrização cutânea. Nosso estudo mostrou que há aumento dos polimorfonucleares e da proliferação vascular. Além disso, há diminuição da formação do colágeno. Assim, a nifedipina tópica pode ter impacto positivo na cicatrização das feridas cutâneas. Estudos adicionais são necessários para confirmar nossos resultados.

## INTRODUCTION

External agents or the patient’s own diseases can cause skin injuries that can challenge healthcare professionals and impose high social, economic, and emotional costs.^1^^-^3

The healing process starts at the time of injury and involves many stages, ranging from hemostasis to tissue regeneration. Angiogenesis occurs in one of these stages and vasodilation is a fundamental mechanism in this process. This stage promotes the arrival of more reparative cells and oxygen to the injury site and can be influenced by intrinsic factors (associated diseases) and extrinsic factors (use of medications).^4^^-^6

Nifedipine (NFD) is a drug that has been used in treatment of hypertensive diseases for more than three decades. It works by promoting arteriolar dilation through blockage of calcium channels in endothelial cells.^7^^-^9 Some authors report that NFD may have some healing benefits in patients with specific chronic wounds related to arteriolar vasospasm, such as hypertensive and scleroderma ulcers, because of better tissue perfusion.^10^^-^12 However, most studies have evaluated the systemic effects of oral NFD. Most of these are case reports, or observations of results using smaller animals (rats), with little relevance for human use because of the great differences between skin types.^13^^,^^14^

Pig skin is considered the best experimental model for comparisons with human skin because of its great histological and functional similarities. These two skin types have similar thickness, sebaceous glands, sweat glands, subcutaneous cellular tissue, and similar hair follicle density. Their regeneration time is around thirty days, and they have a similar biochemical collagen structure. Furthermore, in pig and human skin the healing process occurs through reepithelization, unlike in smaller mammals, where it happens through contraction.^15^^-^18

The aim of this study was to assess the response of wounds treated with topical nifedipine in terms of polymorphonuclear cells, vascular proliferation, and collagen.

## METHODS

### Animals

We used three healthy Pietrain pigs weighing between 15 kg and 20 kg. The animals were housed in appropriate individual stalls with free access to water and standard pig food. Environmental conditions were controlled temperature of 20+/- 2 °C and a dark-light cycle of 12 hours, with ambient relative humidity and noise. The Ethics Committee on Animal Use (CEUA) at the Faculdade de Medicina Nova Esperança (protocol 0024.2015.1) approved this research.

### Anesthesia

The animals initially received a dose of xylazine (1-2 mg/kg) for sedation and analgesia, and a dose of ketamine (2 mg/kg) as a muscle relaxant, followed by orotracheal intubation with a number 7 tube. Anesthesia was maintained with 1% to 2% halogenated isoflurane via inhalation mask and 20 to 50mg of propofol via continuous infusion through the auricular vein, using additional doses according to the animal’s response. The animal was under ventilatory and hemodynamic monitoring throughout the procedure.^19^^,^^20^

### Surgery

After anesthesia, the animals were placed in ventral decubitus position, and an area of 2.5 cm x 2.5 cm was shaved and marked with an appropriate skin marker to standardize wounds. Antisepsis included the use of a chlorhexidine solution followed by alcoholic chlorhexidine 4% on the whole dorsal region, where the fenestrated sterile drape was placed. We proceeded with skin removal until the dorsal muscular fascia was exposed. Hemostasis was achieved with local compression. We cleaned the area with saline solution (SS) 0.9% and applied an NFD dressing to alternate wounds and then wounds were covered with sterile cotton gauze and crepe bandage.

### Flowchart

**Figure 1:**
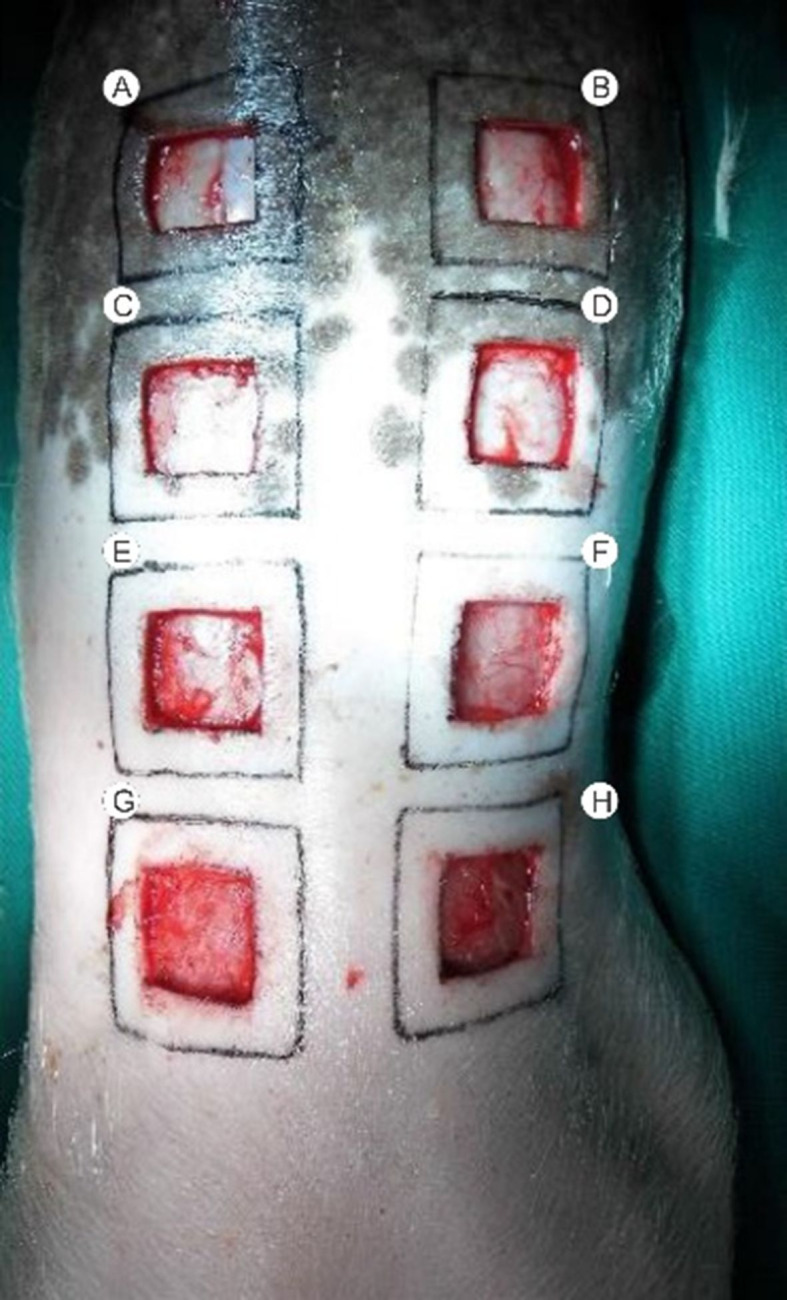
Skin removal down to the dorsal muscular fascia and corresponding letters.

The three animals were placed in separate stalls and identified by numbers 1, 2, and 3. Each wound received a corresponding letter, following a left-to-right and cranial to caudal order (A, B, C, D, E, F, G, and H) (Figure 1). Animal number 1 received topical NFD 1% applied to wounds A, D, E, and H. Animal number 2 received topical NFD 10% applied to wounds B, C, F, and G. Animal number 3 received topical NFD 20% applied to wounds A, D, E, and H. The dressings applied to the other wounds contained only SS 0.9%. Dressings were changed every two days and on data collection days. The 24 wounds were biopsied with a 4 mm punch at the edges on days 1, 3, 7, 14, 21, and 28.

**Figure 2:**
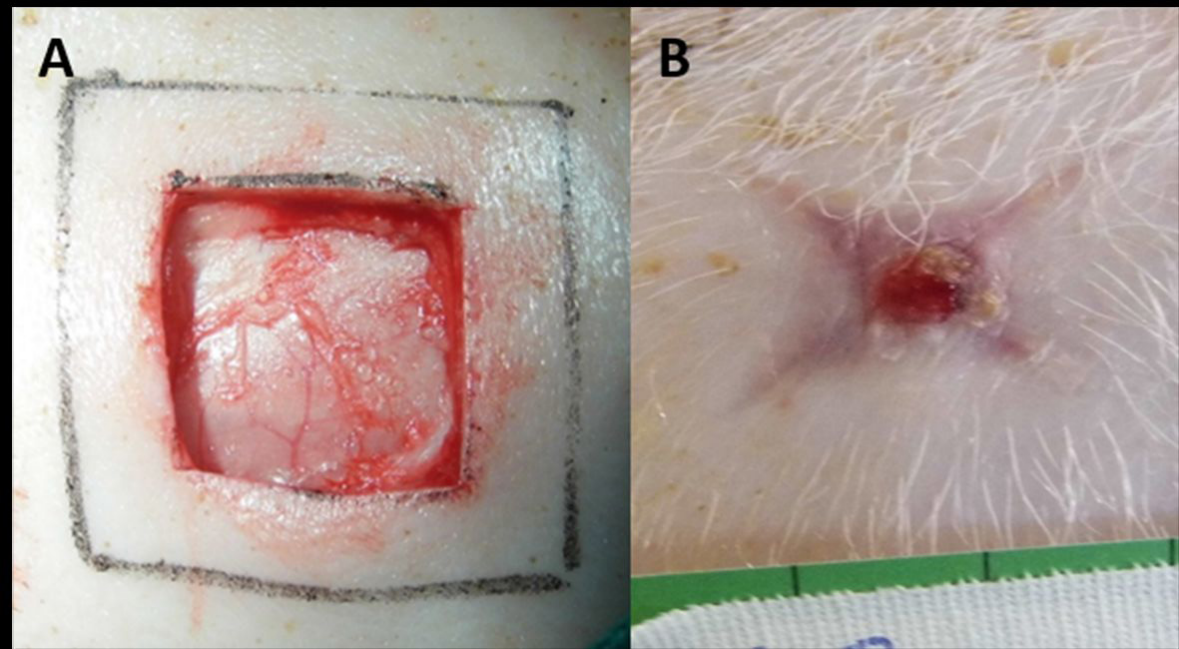
Macroscopic evaluation at the time of wound creation (**A**) and on day 21, with a centimeter rule (**B**).

The wounds were photographed on the days of the biopsies, using a 16-megapixel resolution Panasonic camera, model DMC-LZ30™, with images taken at an angle of 90 degrees to the plane of the wounds at a distance of approximately 30 cm.^21^ To enable correction for any differences in distance, we used a square drawn (4 cm x 4 cm) and overlaid on the wounds (2.5 cm x 2.5 cm) as a reference for the photos taken on day 1. On the other data collection days, a centimeter ruler was glued next to the injuries (Figure 2).

### Microscopic evaluation

All tissue samples were fixed in formalin solution, embedded in paraffin, sectioned serially (5 µm thick), and stained with hematoxylin and eosin. For the purposes of comparison, histological sections were graded at one of four distinct levels according to the presence of agents involved in the healing process.^22^^,^^23^

- Absent (0): not present.
- Mild (1): isolated agents, easily distinguished infiltrate-free areas.
- Moderate (2): irregular presence of agents, many infiltrate-free areas.
- Marked (3): high frequency of agents forming dense aggregates, few infiltrate-free areas.

### Data analysis

A mixed effects logistic model for longitudinal data was used to analyze histological findings. Thus, marked (level 3) production of the different cell types evaluated was considered a response variable (dependent), and the control groups (control, nifedipine 20%) were considered an explanatory variable. Using the control group as the reference, Odds Ratios (OR) were estimated with their respective confidence intervals for the nifedipine groups. The study analyzed the treated group and the control group considering all pigs. Interpretation of an OR in a longitudinal study consists of the odds ratio of the analyzed category compared to the reference category corresponding to the period of analysis, if this association is linear during the whole period. The statistical significance used in this study was 5% (p<0.05) and the software used was SPSS v 21.

## RESULTS

**Figure 3:**
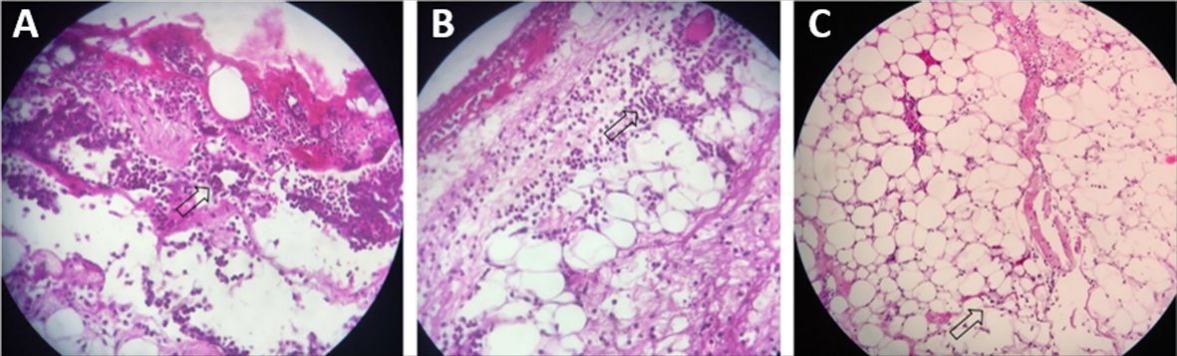
Polymorphonuclear levels. Marked (**A**), Moderate (**B**), and Mild (**C**). See arrows. Hematoxylin-eosin (40 x magnification).

The comparison of wounds treated with NFD 1% versus placebo (pig 1) revealed increased rates of marked polymorphonuclear cell (PMNs) activity (OR 3.5/p = 0.044). There was no statistically significant difference in relation to other parameters (Table 1 and Figure 3).

**Table 1:** Proportion of marked PMN, vascular proliferation, and collagen levels in wounds treated with nifedipine 1% versus placebo.

Data collections	Polymorphonuclear (Marked)		Vascular Proliferation (Marked)		Collagen (Marked)	
	Control	Nifedipine 1%	Control	Nifedipine 1%	Control	Nifedipine 1%
	N (%)	N (%)	N (%)	N (%)	N (%)	N (%)
Day 1	1 (25%)	3 (75%)	0 (0%)	0 (0%)	0 (0%)	0 (0%)
Day 3	0 (0%)	4 (100%)	0 (0%)	0 (0%)	0 (0%)	0 (0%)
Day 7	3 (75%)	3 (75%)	2 (50%)	3 (75%)	0 (0%)	0 (0%)
Day 14	4 (100%)	4 (100%)	3 (75%)	4 (100%)	1 (25%)	1 (25%)
Day 21	1 (25%)	0 (0%)	2 (50%)	3 (75%)	2 (50%)	2 (50%)
Day 28	1 (25%)	3 (75%)	1 (25%)	1 (25%)	4 (100%)	4 (100%)
Nifedipine 1%	OR = 3.5 (CI95%: 1.04 – 11.7)		OR = 1.76 (CI95%: 0.52 – 5.93)		OR = 1.0 (CI95%: 0.12 – 8.62)	
p-value		0.044		0.361		1.000

N – Number; OR – Odds Ratio; CI – Confidence Interval.

Comparison of results for the wounds treated with NFD 10% and for the placebo group (pig 2) was similar to the analysis of NFD 1%, since there was only a statistically significant difference in production of PMNs (OR 11.8/p < 0.001) (Table 2).

**Table 2:** Proportion of marked PMN, vascular proliferation, and collagen levels in wounds treated with nifedipine 10% versus placebo.

**Data collections**	**Polymorphonuclear (Marked)**		**Vascular Proliferation (Marked)**		**Collagen (Marked)**	
	**Control**	**Nifedipine 10%**	**Control**	**Nifedipine 10%**	**Control**	**Nifedipine 10%**
	**N (%)**	**N (%)**	**N (%)**	**N (%)**	**N (%)**	**N (%)**
Day 1	0 (0%)	4 (100%)	0 (0%)	0 (0%)	0 (0%)	0 (0%)
Day 3	0 (0%)	4 (100%)	0 (0%)	0 (0%)	0 (0%)	0 (0%)
Day 7	1 (25%)	2 (50%)	4(100%)	3 (75%)	0 (0%)	0 (0%)
Day 14	0 (0%)	2 (50%)	4(100%)	4 (100%)	0 (0%)	0 (0%)
Day 21	3 (75%)	3 (75%)	3 (75%)	4 (100%)	3 (75%)	0 (0%)
Day 28	1 (25%)	3 (75%)	0 (0%)	3 (75%)	4 (100%)	2 (50%)
Nifedipine 10%	OR = 11.8 (CI95%: 2.99 – 46.2)		OR = 1.78 (CI95%: 0.52 – 6.12)		OR = 0.02 (CI95%: 0.04 – 10.2)	
p-value		<0.001		0.355		0.986

N – Number; OR – Odds Ratio; CI – Confidence Interval.

**Figure 4:**
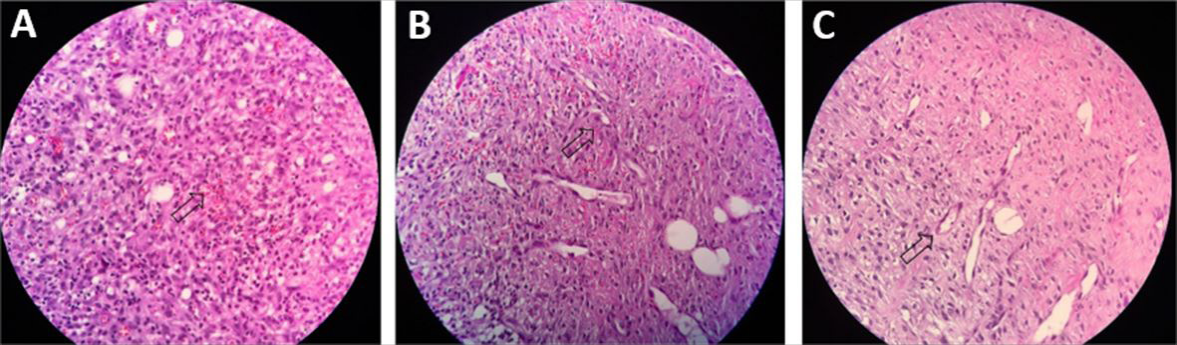
Vascular Proliferation levels. Marked (**A**), Moderate (**B**) and Mild (**C**). See arrows. Hematoxylin-eosin (40 x magnification).

**Figure 5:**
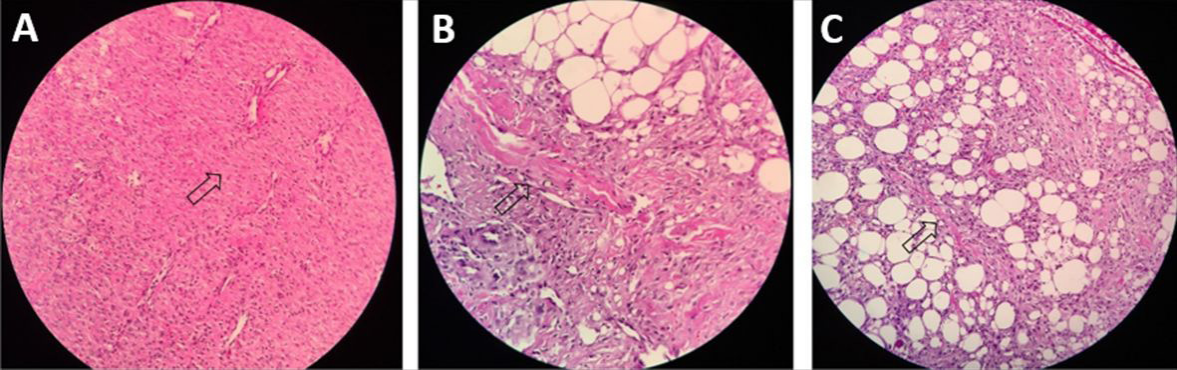
Collagen levels. Marked (**A**), Moderate (**B**) and Mild (**C**). See arrows. Hematoxylin-eosin (40 x magnification).

Comparison between the wounds treated with 20% NFD and the placebo group (pig 3) revealed statistical differences in production of PMNs (OR 22.1/p < 0.001) and marked levels of vascular proliferation (OR 4.84/p = 0.019). There was a statistically significant difference in collagen levels when the groups were compared, but with a lower chance of marked production in the group treated with NFD (OR 0.02/p = 0.005) (Table 3 and Figures 4 and 5).

**Table 3:** Proportion of marked PMN, vascular proliferation, and collagen levels in wounds treated with nifedipine 20% versus placebo.

**Data collections**	**Polymorphonuclear (Marked)**		**Vascular Proliferation (Marked)**		**Collagen (Marked)**	
	**Control**	**Nifedipine 20%**	**Control**	**Nifedipine 20%**	**Control**	**Nifedipine 20%**
	**N (%)**	**N (%)**	**N (%)**	**N (%)**	**N (%)**	**N (%)**
Day 1	0 (0%)	4 (100%)	0 (0%)	0 (0%)	0 (0%)	0 (0%)
Day 3	1 (25%)	4 (100%)	0 (0%)	0 (0%)	0 (0%)	0 (0%)
Day 7	1 (25%)	2 (50%)	3 (75%)	4 (100%)	2 (50%)	0 (0%)
Day 14	2 (50%)	3 (75%)	2 (50%)	3 (75%)	1 (25%)	0 (0%)
Day 21	0 (0%)	4 (100%)	0 (0%)	3 (75%)	4 (100%)	1 (25%)
Day 28	0 (0%)	2 (50%)	0 (0%)	3 (75%)	4 (100%)	1 (25%)
Nifedipine 20%	OR = 22.1 (CI95%: 4.67–104.0)		OR = 4.84 (CI95%: 1.30–18.0)		OR = 0.02 (CI95%: 0.00- 0.28)	
p-value		<0.001		0.019		0.005

N – Number; OR – Odds Ratio; CI – Confidence Interval.

**Figure 6:**
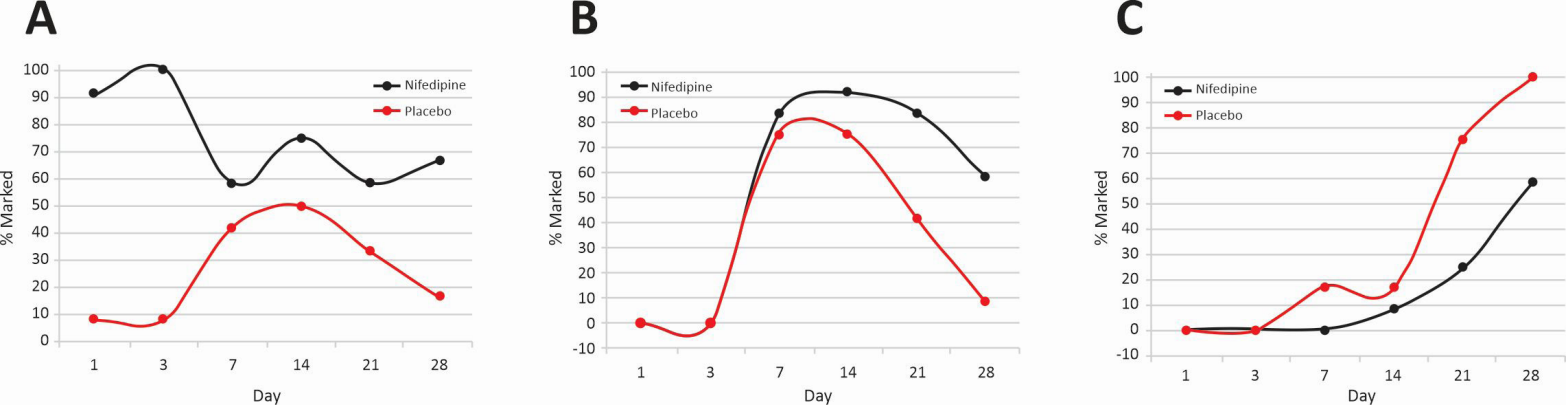
Analysis of the 12 NFD-treated wounds versus the 12 placebo-treated wounds in the three animals. A = polymorphonuclear cells (PMNs); B = vascular proliferation; C = collagen.

Comparative analysis of the 12 NFD-treated wounds versus the 12 placebo-treated wounds for all three animals showed that there were statistically significant increases in PMNs (OR 8.48/p<0.001) and vascular proliferation (OR 2.24/p = 0.019) and statistically significant decreases in collagen levels (OR 0.06/p = 0.006) in the drug-treated wounds (Figure 6).

The model showed an average reduction of 0.188 cm^2^ in the surface area of the wounds every 7 days of observation, but when the groups were compared, the mean difference over time was 0.016 cm^2^, without statistically significant difference between them.

## DISCUSSION

Our study evaluated data on the effects of topical NFD on 24 wounds in three different animals, in a pig model of skin wounds, which is used as a standard for the study of skin wound healing.^15^^-^18

To date, few studies have evaluated the responses of topical NFD using the animal model proposed in this study. Miller et al.^24^ analyzed 16 pigs, each with 4 wounds on the dorsal region, 8 of which were treated with NFD and 8 of which were assigned to a control group. That study did not observe statistical differences between the groups, but we should stress that NFD was administered orally and the only parameter used to evaluate results was wound size.

Ebadi et al.^25^ studied the effect of topical NFD 3% in diabetic and non-diabetic rats. At the end of the experiment, inflammation scored were higher in the diabetic (14.5 vs. 6.5, p<0.005) and non-diabetic (11.3 vs. 5.8, p<0.05) animals treated with topical NFD compared to those treated with placebo. In the diabetic group, use of NFD also interfered in the maturation stage (7.4 vs. 13.6, p<0.05). There was no difference in the proliferation stage in either group.

Healing occurs in three distinct stages (inflammation, proliferation, and maturation), although they occur simultaneously.^4^ In the first stage, inflammatory cells arrive from the remaining vessels in the wound bed. We believe that the vasodilatory effect of NFD caused by blocking entry of calcium into endothelial cells increases PMN levels, as found in our study.

Polymorphonuclear cells have surface adhesion molecules that interact with specific endothelial ligands, crossing the vessel wall through the interendothelial space.^4^^,^^5^^,^^26^^,^^27^ Thus, NFD could facilitate the arrival of these inflammatory cells into the wound bed because of the increased intercellular space caused by vasodilation.

The proliferative stage is when the wound is prepared to be repaired. It includes three distinct stages: angiogenesis, fibroplasia, and epithelization.^4^ Formation of new vessels (angiogenesis) occurs through migration of endothelial cells from preexisting vessels through their intercellular spaces with the help of vasodilation.^5^ The vasodilatory effect of NFD possibly interfered in this healing stage, since a greater occurrence of vascular proliferation was observed in the wounds treated with NFD cream.

Additionally, during the proliferative stage, collagen synthesis from fibroblasts begins and continues until the maturation stage, when there is a balance between production and degradation, and the tensile strength of the scar is maintained by the crosslinks between collagen bundles.^28^ Calcium works by stimulating protein synthesis, especially when it is associated with a cytosolic protein (calmodulin), forming the calcium-calmodulin complex. This compound participates in the release of arachidonic acid from the plasma membrane, enabling production of important healing process stimulators (prostaglandins and leukotrienes), besides acting in protein C kinase production, which acts by stimulating fibroblast proliferation.^29^^,^^30^

We believe that the benefits obtained in the initial phases, with increased inflammatory cells (PMNs) and vascular proliferation, were offset by inhibition of collagen production. Therefore, this ability to stimulate some cells and inhibit others may have contributed to the absence of statistically significant difference in terms of the reduced area in the final stages of the research.

Topical NFD for treating skin wounds is described in medical practice in isolated case reports, without standard concentrations, i.e., it is prescribed off-label.^31^

The present study suggests that topical NFD may interfere in wound healing, since it acts in all three healing stages (inflammatory, proliferative, and maturation), mainly promoting increased inflammatory cells (PMNs), increased vascular proliferation, and decreased collagen.

Future studies are needed to confirm these effects, as well as to identify the best time to use topical NFD to obtain the specific benefits of each healing stage.

The main limitations of our study were: lack of a literature on the specific concentration of the topical NFD, which led us to test three different concentrations, as well as the lack of specific parameters to enable a more reliable comparison of the responses found in the histology. The topical nifedipine may have been absorbed in the wounds treated with the drug, with a likely systemic effect, also affecting the wounds that did not receive the cream directly. Sample size was not calculated and was based on similar studies in the literature and followed the 3 Rs recommendations (Replacement, Reduction and Refinement).^32^ Another limitation was the probable interference of successive biopsies in the healing process.

## CONCLUSIONS

Topical NFD may have an impact on skin wound healing mechanisms, since our study showed that polymorphonuclear cells and vascular proliferation increased. We also demonstrated that collagen formation decreased. Therefore, topical NFD may have a positive impact on skin wound healing. Additional studies are needed to confirm our results.
